# Outcomes of Transposition of Brachiobasilic Arteriovenous Fistula in Two-Stage Technique: A Single-Centre Experience With Literature Review

**DOI:** 10.7759/cureus.9949

**Published:** 2020-08-22

**Authors:** Ketan Mehra, Ramanitharan Manikandan, Lalgudi N Dorairajan, Sreerag Sreenivasan Kodakkattil, Sidhartha Kalra, Rajeev Kumar, Padyala Murali

**Affiliations:** 1 Urology and Renal Transplantation, Jawaharlal Institute of Postgraduate Medical Education and Research, Pondicherry, IND; 2 Urology and Renal Transplantation, Jawaharlal Institute of Post Graduate Medical Education and Research, Pondicherry, IND

**Keywords:** brachiobasilic fistula, arteriovenous fistula, basilic vein, two-stage arterivenous fistula, haemodialysis

## Abstract

Introduction

Arteriovenous fistulae (AVF) are considered a better option for long-term dialysis access. The distal radiocephalic AVF is the most preferred followed by proximal radiocephalic, brachiocephalic and brachiobasilic AVFs (BBAVF) with basilic vein transposition. In case of failure of AVF at other anatomical locations, BBAVF may improve the outcomes for patients needing dialysis for long term. The two-stage technique of BBAVF has easier dissection and lesser devascularisation risk. The disadvantages are need for two interventions and delay in maturation.

Materials and Method

It was a retrospective observational study including 42 patients who underwent transposition of BBAVF as two-stage procedure from June 2014 to July 2018. The data recorded were demographic characteristics, such as median age, gender, dialysis status at AVF creation and operative duration. Complications like postoperative limb oedema, bleeding and thrombosis of AVF were recorded. Patency and access outcome of AVF were documented at three-month follow-up.

Results

Among 42 patients, 27 (64.3%) were males. The median age was 50 years. Around 14% of patients had minor complications like oedema. Eight (19%) patients needed re-exploration due to bleeding or thrombosis. The early access failure rate that is a failure before discharge was 4.7%. The patency rate at three months was 90.5%, but the primary functional rate was 74%.

Conclusion

Transposition of BBAVF as a two-step technique is associated with reasonable patency rate and primary functional rate. The related complications were low, and a good number of fistulae could be saved with timely intervention.

## Introduction

In patients with end-stage renal disease (ESRD), arteriovenous fistulae (AVF) are a better option than central venous catheters (CVC) or arteriovenous grafts (AVG) as a strategy for long-term dialysis access [1]. The reported incidence of complications like thrombosis, stenosis and infections is less with AVF as compared to CVC and AVG [1-3]. However, AVFs have a high failure rate and approximately 20%-50% of them fail to mature and become unsuitable for haemodialysis [4-6]. Based on the location, the National Kidney Foundation Kidney Disease Outcomes Quality Initiative (NKF KDOQI) guidelines recommend the preferential sites for AVF creation in the following order: the distal radiocephalic AVF being the most preferred followed by a proximal AVF which could be either radiocephalic, brachiocephalic or brachiobasilic AVFs (BBAVF) with basilic vein transposition [7]. The advantages of a distal radiocephalic AVF over proximal AVF include a higher patency rate and a fewer requirement for secondary procedures. However, its main drawback is the high failure rate to mature [8]. Besides, the choice of anatomical site for creating AVF is aided by ultrasound-guided vascular mapping, which is a better predictor for a successful outcome in terms of AVF maturation [9,10].

In 1976, Dagher et al. were the first to describe the technique of fashioning a brachiobasilic fistula by using the basilic vein in an end-to-side fashion with the brachial artery for haemodialysis access [11]. Subsequently, the surgical technique has evolved over the years to improve the outcomes. The two primary methods to make the BBAVF more superficial and easy to cannulate during haemodialysis are the elevation technique and transposition technique [12,13]. In the elevation technique, the deep fascia is reconstructed without the mobilisation of the vein [12]. In contrast, the vein is mobilised superficially in an anterolateral position after creating a skin flap in the transposition technique [13]. The BBAVF can be created either by a one-stage or a two-stage technique. In a one-stage procedure, the vein is mobilised and anastomosis is fashioned in a single operation. It provides an earlier functional AVF [13]. In the two-stage procedure, initially, the fistula is created in the original anatomical position, and the matured AVF is transposed superficially and laterally in the second stage. The presumed advantage is the lesser risk of devascularisation in dissecting and mobilising the already arterialised vein [14]. However, the downside of this technique is the need for two surgical interventions and the subsequent delay in providing access for haemodialysis. The primary objective of this study is to determine the outcomes of BBAVF transposition performed as a two-stage procedure.

## Materials and methods

The present study was conducted at a tertiary health care centre in South India. It was a retrospective study and involved evaluation of data of 42 patients in whom BBAVF transposition was performed as a two-staged procedure at our centre during the study period from June 2014 to July 2018. In all the patients, BBAVF was performed as a secondary or tertiary vascular access site after failed access at distal locations. During the first stage, the end of the basilic vein was anastomosed to the side of the brachial artery. The second stage of basilic vein transposition was performed six weeks after the first procedure.

**Figure 1:**
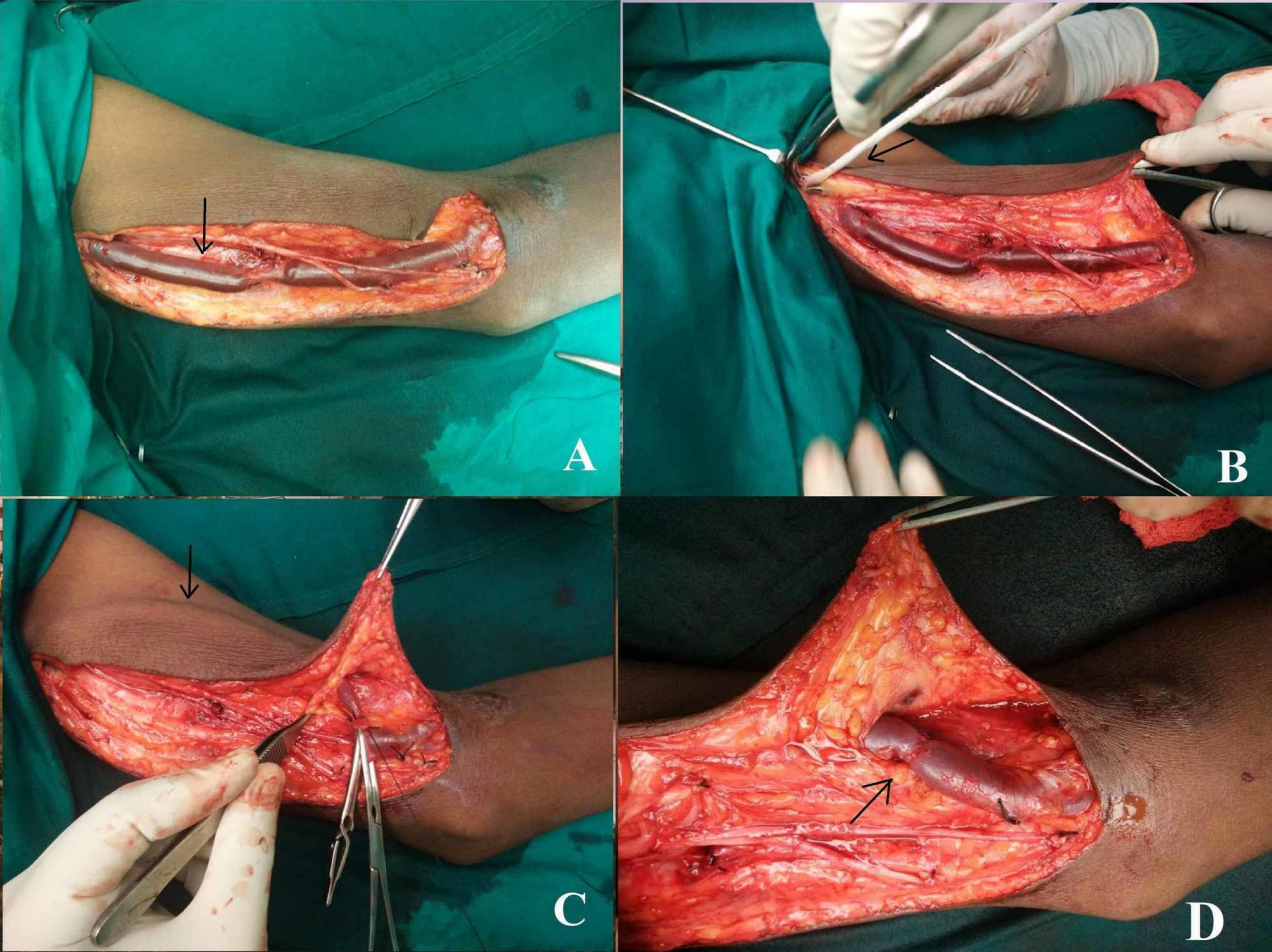
Showing steps of brachiobasilic transposition. (A) Incision along the medial aspect of the arm. The arrow showing basilic vein after dissection. (B) Subcutaneous tunnel made by passing a passer indicated by the arrow. (C) Transposed vein (black arrow). (D) Brachiobasilic anastomosis (black arrow).

The maturation of the fistula was closely monitored in the intervening period by regular clinical examination. During the second-stage operation, a longitudinal incision was made over the medial aspect of the arm following the course of the basilic vein. The basilic vein was dissected and disconnected off the BBAVF. It was transposed superficially by creating a separate tunnel in the subcutaneous plane and re-anastomosed to the brachial artery (Figure 1).

All patients received anticoagulation in the form of intravenous heparin intraoperatively before anastomosis of the vessels. Postoperatively, the decision for heparin administration was decided by the surgeon depending on the functioning of the AVF and haemostasis. A suction drain was placed under the flap. The various parameters that were recorded during and after second-stage procedures include demographic characteristics like median age, gender, dialysis status at AVF creation, operative duration, postoperative limb oedema, bleeding, thrombosis of the AVF and the access outcome. The complications were classified by the modified Clavien-Dindo method of classification (Table 1).

**Table 1:** Complications as per Clavien-Dindo classification

Grade	Number (%)	Cases
II	6 (14.3)	Ipsilateral upper limb oedema (n=6). Surgical site infection - antibiotics and dressing (n=4)
IIIa	8 (19)	Bleeding requiring re-exploration under local anaesthesia (n=2). Thrombectomy under local anaesthesia (n=6)

At three-month follow-up, the patency and access outcome of AVF were documented. The access was considered mature and successful if cannulation was possible for dialysis with a flow rate of at least 300 mL/min. The patient who underwent any kind of intervention post-second stage was recorded.

## Results

A total of 42 patients underwent transposition of BBAVF as a two-stage procedure. The median age of the patients was 50 years (range 27-72 years). There were 15 (35.7%) females and 27 (64.3%) males in our series. A total of 30 (71.4%) patients were already on dialysis at the time of the creation of AVF on temporary cannula (Table 2).

**Table 2:** Demographic data

Variables	Data
Age in years, median (range)	50 (27-72)
Male (%)	27 (64.3)
Female (%)	15 (35.7%)
Body mass index, median (range)	22 (18-28)
Patient already on haemodialysis (%)	30 (71.4)

All these patients had an already failed AVF surgery at other sites (distal or proximal). The median numbers of previous failed excess sites were 2 (0-4). All patients underwent brachiobasilic anastomosis in an end-to-side fashion in the first stage. The median follow-up of the patients was three months (one to seven months). All BBAVFs were created under local anaesthesia with sedation. The median operative time was 218 minutes (180-240 minutes). The median blood loss was 140 mL (50-250 mL). The median length of hospital stay was three days.

Among the 42 patients of transposed BBAVF, 6 (14.3%) patients developed postoperative ipsilateral limb oedema, which was managed conservatively. Among these six patients, four had surgical site infection, which was managed by dressing and antibiotics. Two (4.7%) patients required re-exploration to control bleeding in the immediate postoperative period. Postoperative thrombosis of draining vein occurred in six (14.3%) patients. This thrombotic event occurred within 24 hours of the second-stage procedure. In four (66%) patients, the fistula was successfully salvaged by an immediate thrombectomy. Forty (95.2%) patients had a patent fistula at the time of discharge from the hospital with early access failure in two (4.7%) patients due to thrombus formation which could not be salvaged even after immediate surgical exploration. At the time of follow-up, 38 (90.5%) patients were alive at three months with a patent transposed BBAVF. Four (9%) patients never received dialysis as they underwent renal transplant before the need for dialysis initiation. Three (7%) female patients had a primary access failure as cannulation for dialysis was not successful at three months post-transposition due to reduced flow rates. Overall, 31 (73.8%) patients had a mature access site and received dialysis through the functional transposed BBAVF.

## Discussion

Maya et al. retrospectively reviewed the clinical outcomes of upper arm vascular access in 678 patients with AVF. Their series comprised of 322 brachiocephalic fistulas, 67 brachiobasilic fistulas, and 289 AVGs [15]. The reported incidence of primary access failure was 15%-18% in the transposed BBAVF group and a significantly lower primary access failure rate in males as compared to females. Moreover, there was no difference in the successful primary access rate of graft versus transposed BBAVF. Moreover, interventions per year to salvage AVF were lower for brachiobasilic and brachiocephalic fistulas in comparison to AVGs (0.84, 0.82 and 1.87, respectively, P < 0.001).

In one of the most extensive studies reported in the literature, Vrakas et al. compared the outcomes of one-stage and two-stage BBAVF involving 149 brachiobasilic transpositions in 141 patients [13]. A total of 65 patients underwent one-stage surgery and 84 had two-stage surgery. This study reported no difference in primary failure rate between the two groups, but in multivariate analysis, the one-stage procedure had 3.2 times more chances of overall failure. The likelihood of failure was 2.7 times more in males, but the difference was not statistically significant. Five (16%) patients developed early complications in the one-stage group (thrombosis-2, haematoma-2, steal phenomenon-1). However, only one (2%) patient developed a haematoma in the two-stage procedure.

In another study by Shibutani et al., 24 patients underwent single-stage transposition of BBAVF [16]. The mean operative time was 136 (90-210) minutes. There was oedema of the upper extremity in all patients, which was managed conservatively. Two (8%) patients had a surgical site infection. Four (16%) patients developed with thrombotic occlusion of AVF. The mean follow-up period was 18 (3-40) months. The reported primary patency rates at one and two years of follow-up were 89.7% and 69.0%, respectively. The secondary patency rates were 95.7% and 73.6%, respectively.

Bashar et al. performed a meta-analysis of eight published studies comparing the outcomes of one-stage and two-stage BBAVFs [17]. The study included the data of 849 patients with 859 fistulae with one-stage and two-stage approaches in 366 (42.6%) and 493 (57.4%) patients, respectively. The authors reported no significant difference between the two techniques in terms of the rate of successful maturation and patency rates. The incidence of wound infection, haematoma formation and steal syndrome was similar between the two groups.

In another study reported by Veeramani et al., transposition of BBAVF was performed in a single stage by a novel small incision technique to reduce the complications like limb oedema and also to avoid extensive tissue dissection [18]. The authors reported a primary patency rate of 71.42% at one year. However, the incidence of limb oedema was 14.2%, which was comparable to our study. Approximately 21.4% of patients in their study required re-exploration due to bleeding, haematoma or thrombosis similar to our study.

In another meta-analysis by Sheta et al., 37 studies on one-stage versus two-stage BBAVF were evaluated. The one-year primary patency rates and incidence of complications were equivalent between the two procedures [19]. However, the secondary patency rate at one year was higher in the two-stage technique (79% versus 85%).

Kakkos et al. reported that the incidence of complications was significantly higher in one-stage BBAVF [20]. In their series, the incidence of venous hypertension, wound haematoma and overall complications in the one-stage procedure was 17%, 13% and 43% and in the two-stage procedure was 4%, 3% and 11%, respectively. Besides, the meantime for maturation in one-stage and two-stage technique was 68 days and 132 days, respectively. Generally, all published studies report a higher maturation rate with two-stage procedure, albeit with a longer time to cannulation [21-23].

Ghaffarian et al. compared one-stage and two-stage procedure, and concluded that two-stage procedure was more cost-effective as it had lower quality-adjusted life years (QALY) [24]. The cost for two-stage technique of BBAVF was $4,730 in comparison to one-stage technique that cost $4,412. However, with secondary patency outcomes into consideration, the two-stage technique was more cost-effective than the one-stage technique (3.74 QALYs for two-stage technique versus 3.32 QALYs for one-stage technique) during the five-year period.

As per our institutional protocol, patients who require vascular access for haemodialysis, distal radiocephalic, proximal radiocephalic and brachiocephalic AVFs are considered in that order. In cases where all the above options were exhausted, the patient was considered for two-stage BBAVF. Around 14% of patients had minor complications like oedema. Eight (19%) patients needed re-exploration due to bleeding or thrombosis. The early access failure rate, that is a failure before discharge, was 4.7%. The patency rate at three months was 90.5%, but the primary functional rate was 74%.

There were some limitations of this study. Firstly, it was a retrospective observational study. Secondly, the sample size was smaller. A comparative study would yield better results comparing one-stage versus two-stage, grafts versus AVF and BBAVF versus brachiocephalic AVF.

## Conclusions

The transposition of BBAVF as a part of two-stage technique of fashioning is associated with reasonable patency rate and primary functional rate. The related complications were low, and a good number of fistulae could be saved with timely intervention.

## References

[1] Bashar K, Healy D, Browne LD (2014). Role of far infra-red therapy in dialysis arterio-venous fistula maturation and survival: systematic review and meta-analysis. PLoS One.

[2] Frankel A (2006). Temporary access and central venous catheters. Eur J Vasc Endovasc Surg.

[3] Spergel LM, Ravani P, Roy-Chaudhury P, Asif A, Besarab A (2007). Surgical salvage of the autogenous arteriovenous fistula (AVF). J Nephrol.

[4] (1997). NKF-DOQI clinical practice guidelines for vascular access. National Kidney Foundation-Dialysis Outcomes Quality Initiative. Am J Kidney Dis.

[5] Lynch JR, Mohan S, McClellan WM (2011). Achieving the goal: results from the Fistula First Breakthrough Initiative. Curr Opin Nephrol Hypertens.

[6] Ethier JH, Lindsay RM, Barre PE, Kappel JE, Carlisle EJ, Common A (1999). Clinical practice guidelines for vascular access. Canadian Society of Nephrology. J Am Soc Nephrol.

[7] (2020). 2006 Updates Clinical Practice Guidelines and Recommendations. https://www.kidney.org/sites/default/files/docs/12-50-0210_jag_dcp_guidelines-hd_oct06_sectiona_ofc.pdf.

[8] Sultan S, Hynes N, Hamada N, Tawfick W (2012). Patients on hemodialysis are better served by a proximal arteriovenous fistula for long-term venous access. Vasc Endovascular Surg.

[9] Ilhan G, Esi E, Bozok S (2013). The clinical utility of vascular mapping with Doppler ultrasound prior to arteriovenous fistula construction for hemodialysis access. J Vasc Access.

[10] Heye S, Fourneau I, Maleux G, Claes K, Kuypers D, Oyen R (2010). Preoperative mapping for haemodialysis access surgery with CO(2) venography of the upper limb. Eur J Vasc Endovasc Surg.

[11] Dagher F, Gelber R, Ramos E, Sadler J (1976). The use of basilic vein and brachial artery as an A-V fistula for long term hemodialysis. J Surg Res.

[12] Hossny A (2003). Brachiobasilic arteriovenous fistula: different surgical techniques and their effects on fistula patency and dialysis-related complications. J Vasc Surg.

[13] Vrakas G, Defigueiredo F, Turner S, Jones C, Taylor J, Calder F (2013). A comparison of the outcomes of one-stage and two-stage brachiobasilic arteriovenous fistulas. J Vasc Surg.

[14] Francis DM, Lu Y, Robertson AJ, Millar RJ, Amy J (2007). Two-stage brachiobasilic arteriovenous fistula for chronic haemodialysis access. ANZ J Surg.

[15] Maya ID, O'Neal JC, Young CJ, Barker-Finkel J, Allon M (2009). Outcomes of brachiocephalic fistulas, transposed brachiobasilic fistulas, and upper arm grafts. Clin J Am Soc Nephrol.

[16] Shibutani S, Obara H, Ono S, Kakefuda T, Kitagawa Y (2013). Transposed brachiobasilic arteriovenous fistula. Ann Vasc Dis.

[17] Bashar K, Healy DA, Elsheikh S (2015). One-stage vs. two-stage brachio-basilic arteriovenous fistula for dialysis access: a systematic review and a meta-analysis. PLoS One.

[18] Veeramani M, Vyas J, Sabnis R, Desai M (2010). Small incision basilic vein transposition technique: a good alternative to standard method. Indian J Urol.

[19] Sheta M, Hakmei J, London M, Wooster M, Aruny J, Ross J, Illig KA (2020). One- versus two-stage transposed brachiobasilic arteriovenous fistulae: a review of the current state of the art. J Vasc Access.

[20] Kakkos SK, Haddad GK, Weaver MR, Haddad RK, Scully MM (2010). Basilic vein transposition: What is the optimal technique?. Eur J Vasc Endovasc Surg.

[21] Kakkos SK, Tsolakis IA, Papadoulas SI (2015). Randomized controlled trial comparing primary and staged basilic vein transposition. Front Surg.

[22] Ozcan S, Gür AK, Yener AU, Odabaşi D (2013). Comparison of one- and two-stage basilic vein transposition for arterio-venous fistula formation in haemodialysis patients: preliminary results. Cardiovasc J Afr.

[23] Agarwal A, Mantell M, Cohen R, Yan Y, Trerotola S, Clark TW (2014). Outcomes of single-stage compared to two-stage basilic vein transposition fistulae. Semin Dial.

[24] Ghaffarian AA, Griffin CL, Kraiss LW, Sarfati MR, Brooke BS (2018). Comparative effectiveness of one-stage versus two-stage basilic vein transposition arteriovenous fistulas. J Vasc Surg.

